# Applying Sodium Carbonate Extraction Mass Spectrometry to Investigate Defects in the Mitochondrial Respiratory Chain

**DOI:** 10.3389/fcell.2022.786268

**Published:** 2022-03-01

**Authors:** David R. L. Robinson, Daniella H. Hock, Linden Muellner-Wong, Roopasingam Kugapreethan, Boris Reljic, Elliot E. Surgenor, Carlos H. M. Rodrigues, Nikeisha J. Caruana, David A. Stroud

**Affiliations:** ^1^ Department of Biochemistry and Pharmacology, Bio21 Molecular Science and Biotechnology Institute, University of Melbourne, Parkville, VIC, Australia; ^2^ The Royal Children’s Hospital, Murdoch Children’s Research Institute, Parkville, VIC, Australia; ^3^ Department of Biochemistry and Molecular Biology, Monash Biomedicine Discovery Institute, Monash University, Clayton, VIC, Australia; ^4^ The Walter and Eliza Hall Institute of Medical Research, Parkville, VIC, Australia; ^5^ Baker Heart and Diabetes Institute, Melbourne, VIC, Australia; ^6^ Institute for Health and Sport (IHES), Victoria University, Melbourne, VIC, Australia

**Keywords:** mitochondria, proteomic analyses, membrane protein, OXPHOS (oxidative phosphorylation), respiratory chain assembly, carbonate extraction

## Abstract

Mitochondria are complex organelles containing 13 proteins encoded by mitochondrial DNA and over 1,000 proteins encoded on nuclear DNA. Many mitochondrial proteins are associated with the inner or outer mitochondrial membranes, either peripherally or as integral membrane proteins, while others reside in either of the two soluble mitochondrial compartments, the mitochondrial matrix and the intermembrane space. The biogenesis of the five complexes of the oxidative phosphorylation system are exemplars of this complexity. These large multi-subunit complexes are comprised of more than 80 proteins with both membrane integral and peripheral associations and require soluble, membrane integral and peripherally associated assembly factor proteins for their biogenesis. Mutations causing human mitochondrial disease can lead to defective complex assembly due to the loss or altered function of the affected protein and subsequent destabilization of its interactors. Here we couple sodium carbonate extraction with quantitative mass spectrometry (SCE-MS) to track changes in the membrane association of the mitochondrial proteome across multiple human knockout cell lines. In addition to identifying the membrane association status of over 840 human mitochondrial proteins, we show how SCE-MS can be used to understand the impacts of defective complex assembly on protein solubility, giving insights into how specific subunits and sub-complexes become destabilized.

## 1 Introduction

Mitochondria are double membrane-bound eukaryotic organelles that perform critical cellular functions including producing the bulk of cellular energy, acting as hubs for synthesis of various biomolecules, and driving the apoptotic response. Mitochondria produce the majority of the cell’s ATP through oxidative phosphorylation (OXPHOS), which involves five multi-protein complexes (complexes I-IV and the F_1_-F_0_ ATP Synthase, or complex V) found within the inner mitochondrial membrane (IMM) (Hatefi, 1985; Rich and Maréchal, 2010; Hock et al., 2020). Mitochondria evolved from endosymbiotic prokaryotes, and therefore have their own genome, known as mitochondrial DNA (mtDNA), and their own ribosomes, known as mitoribosomes. Most protein coding genes in mtDNA were lost or transferred to the nucleus early in eukaryotic evolution, and numerous machineries have evolved to import and process these >1,000 proteins (Adams and Palmer, 2003; Roger et al., 2017; Rath et al., 2021). Following synthesis in the cytosol, nuclear encoded proteins are imported into the mitochondria through translocases of the inner and outer membranes, TIM and TOM respectively (Jackson et al., 2018; Pfanner et al., 2019). In humans, 13 proteins are encoded by mtDNA, all hydrophobic transmembrane subunits of the OXPHOS complexes that are translated on mitoribosomes and co-translationally inserted into the IMM (Ott and Herrmann, 2010; Thompson et al., 2018).

Complexes I-IV act as an electron transport chain with electrons from metabolic sources such as the tricarboxylic acid (TCA) cycle passing from complex to complex in a series of redox reactions. The energy from this process is used to pump protons from the mitochondrial matrix to the intermembrane space (IMS), creating a proton gradient used by complex V to synthesize ATP (Jonckheere et al., 2012). Mitochondrial respiratory complexes I, III and IV are found together in higher-order complexes, so called supercomplexes (SCs), that have well defined stoichiometry (Schagger and Pfeiffer, 2000; Letts et al., 2016; Wu et al., 2016; Guo et al., 2017). These include complex I and III (I/III_2_), complex III and IV (III_2_/IV), and complex I, III and IV (I/III_2_/IV), the latter referred to as the respirasome and considered the dominant form.

Complex III (CIII) or the cytochrome *bc*_1_ complex is at the center of the OXPHOS system, using electrons received from complexes I, II and other sources via ubiquinone to reduce cytochrome *c*, while also pumping protons into the IMS (Crofts et al., 2008; Fernandez-Vizarra and Zeviani, 2015). Mammalian CIII forms an obligate homodimer, with each subunit composed of 9 nuclear encoded proteins and one mtDNA-encoded protein, cytochrome *b* (MT-CYB) (Schagger et al., 1986). Complex III biogenesis begins with the synthesis of mtDNA-encoded MT-CYB on mitoribosomes and its co-translational insertion into the IMM. Two small membrane bound subunits UQCRB and UQCRQ then associate with MT-CYB (Hildenbeutel et al., 2014), while in parallel to this, peripherally associated subunits UQCRC1 and UQCRC2 form a tetramer of two UQCRC1-UQCRC2 dimers which is thought to coalesce on the nascent MT-CYB assembly along with additional membrane bound subunits CYC1, UQCR10 and UQCRH to yield a dimeric intermediate of CIII called pre-CIII_2_ (Stephan and Ott, 2020). Finally, UQCR11 and catalytic subunit UQCRFS1 and incorporated to form the mature, active complex (Smith et al., 2012; Fernandez-Vizarra and Zeviani, 2018; Ndi et al., 2018). Although complexes I and IV are structurally independent from CIII, complete complex III assembly is critical for their biogenesis (Acin-Perez et al., 2004; Protasoni et al., 2020). Although the molecular details of this necessity are not yet fully understood, loss of CIII leads to inhibition of the final step of Complex I assembly; addition of the catalytic NADH dehydrogenase module (N-module) (Protasoni et al., 2020). Mutations in MT-CYB and 5 of the 9 nuclear encoded subunits in Complex III, along with mutations in many Complex I and IV subunits and assembly factors required for the biogenesis of OXPHOS complexes, impact complex and result in mitochondrial disease, a debilitating metabolic disorder affecting at least 1 in 5,000 live births (Fernandez-Vizarra and Zeviani, 2015; Gorman et al., 2016; Hock et al., 2020). For the majority of mitochondrial diseases there are no effective treatments, thus a nuanced understanding the molecular impact of dysfunctional assembly will be critical to the future development of therapeutics (Gorman et al., 2016).

Sodium carbonate extraction is a ∼30 year old experimental technique used to determine whether a specific protein is an integral membrane protein, peripherally associated with the membrane, or a soluble protein. In the original method, cellular isolates containing membranes are solubilized in 0.1 M sodium carbonate, pH 11.5 (Fujiki et al., 1982a; Fujiki et al., 1982b). The alkaline carbonate solution disrupts protein-protein interactions without disrupting the membrane. Ultracentrifugation is then used to generate a pellet fraction, containing integral membrane proteins, and a supernatant fraction containing soluble and membrane-associated proteins. These fractions can then be analyzed by immunoblotting with specific antibodies.

The mitochondrial inner membrane is one of the most densely packed eukaryotic membranes with a high protein: lipid ratio. As a result of this, the transmembrane domains (TMDs) of mitochondrial membrane proteins are less hydrophobic compared to ER membrane proteins (von Heijne, 1986). This, along with the unique lipid content of mitochondrial membranes may explain the observation that mitochondrial proteins with single, moderately hydrophobic TMDs are susceptible to carbonate extraction at pH 11.0 (Kim et al., 2015). Thus, sodium carbonate extraction alone may yield false negatives as to the presence of TMDs in mitochondrial proteins, and that proteins may be present in both the pellet and supernatant fractions in different proportions, depending on their level of membrane integration (Fujiki et al., 1982a). One approach to overcome this is the use of multiple carbonate solutions with increasingly lower pH, as at lower pH peripheral membrane proteins are partially retained in the pellet fraction (Lytovchenko et al., 2014).

Here we describe a technique combining sodium carbonate extraction with quantitative mass spectrometry (SCE-MS) applicable to routine use in molecular investigations. We used SCE-MS to generate membrane association profiles for over 840 mitochondrial proteins in human embryonic kidney (HEK293T) cells, grouping them into 6 distinct clusters based on their resistance to sodium carbonate extraction across multiple pH conditions. By coupling SCE-MS with stable isotope labelling of amino acids in cell culture (SILAC), we were able to track changes in the membrane integration of mitochondrial proteins in gene-edited knockout cell-lines lacking different CIII subunits. We find that loss of single pass membrane-spanning subunit UQCR10 leads to defective complex assembly. SCE-MS revealed that while the majority of CIII subunits remained membrane associated, peripherally associated core-module subunits UQCRC1 and UQCRC2 became more soluble, consistent with the destabilization and detachment of the core-module from the membrane bound CIII intermediate. Additionally, we found that arrest of CIII assembly, either through loss of UQCR10 or UQCRC1, leads to partial loss of the Complex I N-module as has been previously shown in CIII deficient cells (Protasoni et al., 2020). Using SCE-MS we found that while a population of N-module remained associated with Complex I, N-module subunits had increased solubility relative to other CI subunits, suggesting the modules attachment to the complex becomes unstable in the presence of a structural CIII defect. Thus, we see SCE-MS as a novel and unbiased means to assess the destabilization of protein complexes; a concept often cited to describe the impact of an assembly defect, but generally without a clear molecular definition.

## 2 Materials and Methods

### 2.1 Tissue Culture and Generation of Knockout Cell Lines

HEK293T cells were cultured in Dulbecco’s Modified Eagle’s medium (DMEM High Glucose), supplemented with 10% (v/v) fetal calf serum (FCS; CellSera, Rutherford, NSW, Australia), penicillin/streptomycin (Thermo Fisher Scientific, Scoresby, VIC, Australia) and 50 μg ml^−1^ uridine (Sigma-Aldrich, North Ryde, NSW, Australia) at 37°C under an atmosphere of 5% CO_2_. Guide (g)RNA sequences for CRISPR-Cas9 gene editing were designed using the CHOPCHOP software (Labun et al., 2019) and corresponding oligonucleotides were cloned into pSPCas9(BB)-2A-GFP (PX458) plasmid (a gift from F. Zhang;RRID:Addgene_48138) as previously described. The gRNA sequences used were 5′-CGA​CTG​CAG​CTG​GAA​GAT​GG for UQCRC1^KO^ and 5′-TGG​ACT​GTG​AAG​AAA​CAT​GG for UQCR10^KO^. HEK293T cells were transfected with constructs using Lipofectamine LTX (Thermo Fisher Scientific) according to manufacturer’s instructions. Single GFP-positive cells were sorted into 96 well plates and expanded for screening. To verify mutations caused by CRISPR/Cas9, genomic DNA of positive clones was extracted using Quick-DNA kit (Zymo Research, Irvine, CA) according to manufacturer’s specifications. Screening primers designed by the CHOPCHOP software were used to amplify target regions that were cloned into the pGEM4Z plasmid (Yanisch-Perron et al., 1985) for M13 primed Sanger sequencing as described previously (Stroud et al., 2016). (Stroud et al., 2016). UQCRC1^KO^ clone 1 contained the following mutations c.[1-6T_11del]; [1-3A_4del] while clone 2 contained c.[1-126T_69+66delins]; [3G_4del] c.[1-126T_69+65delins]. UQCR10^KO^ clone 1 contained the following mutations c.[1-217G_30del] while UQCR10^KO^ clone 2 contained c.[1-27G_8del]; [1-11T_14del]; [1-28G_128delins]. All mutations disrupted the start codon and as such no protein product is predicted.

### 2.2 SDS-PAGE, BN-PAGE and Western Blotting

SDS-PAGE was performed using samples solubilized in LDS sample buffer (Thermo Fisher Scientific) with the 100 mM DTT and separated on Invitrogen Bolt Bis-Tris 4–12% protein gels according to manufacturer’s conditions. BN-PAGE was performed using mitochondrial proteins solubilized in 1% (w/v) digitonin buffer as previously described (Wittig et al., 2006; Lazarou et al., 2007). Mitochondria were isolated as previously described (Johnston et al., 2002) with protein concentration determined using the Pierce Protein Assay Kit (Thermo Fisher Scientific). Detergent solubilized complexes were separated on Invitrogen NativePAGE Bis-Tris gels (3–12%) according to manufacturer’s instructions. Western blotting of SDS-PAGE gels was undertaken using the Invitrogen iBlot2 Dry Blotting System using PVDF based stacks as per manufacturer’s instructions. Western transfer of BN-PAGE gels onto PVDF (Merck, Bayswater, VIC, Australia) was performed using the Invitrogen Mini Blot Module transfer system as per manufactures recommendations. Immunoblots were developed using horseradish peroxidase-conjugated mouse and rabbit secondary antibodies (Cell Signaling Technology, Danvers, MA) and ECL chemiluminescent substrates (Bio-Rad, Gladesville, NSW, Australia) and visualized using a ChemiDoc XRS+ gel documentation system (Bio-Rad). Commercial antibodies were acquired for COXI (Abcam Cat# ab14705, RRID:AB_2084810), COXII (Thermo Fisher Scientific Cat# A-6404, RRID:AB_221584), COX4 (Abcam Cat# ab110261, RRID:AB_10862101), CYC1 (Sigma-Aldrich Cat# HPA001247, RRID:AB_1078602), CYCS (BD Biosciences Cat# 556433, RRID:AB_396417), NDUFV1 (Proteintech Cat# 11238-1-AP, RRID:AB_2149040), SDHA (Abcam Cat# ab14715, RRID:AB_301433), TIMM50 (Proteintech Cat# 22229-1-AP, RRID:AB_2879039), UQCRC1 (Abcam Cat# ab110252, RRID:AB_10863633), UQCRFS1 (Abcam Cat# ab14746, RRID:AB_301445), UQCRH (Abcam Cat# ab134949, RRID:AB_2800504), and UQCRQ (Abcam Cat# ab110255, RRID:AB_10865309). The generation of polyclonal antibodies for MT-HSP70 (Stroud et al., 2013), VDAC2 (Ma et al., 2014), NDUFAF1 (Dunning et al., 2007) and NDUFA9 (Lazarou et al., 2007) has been previously described. In-gel activity assay was performed as previously described (Zerbetto et al., 1997).

### 2.3 Sodium Carbonate Extraction for SDS-PAGE and BN-PAGE

Mitochondria were isolated from HEK293T cells were pelleted at 12,000 x g for 5 min at 4°C before being resuspended in 100 mM Na_2_CO_3_, pH 9.5 or 11.5 and incubated on ice for 30 min. Half the volume of each sample was reserved for a “Total” fraction. The remainder was centrifuged at 125,000 x g for 30 min at 4°C to generate “Pellet” and “Supernatant” fractions. The pellet fraction was solubilized for SDS-PAGE as described above. Total and supernatant fractions were made up to a final concentration of 15% trichloroacetic acid (TCA) and incubated on ice for 30 min before centrifugation at 20,000 x g for 10 min at 4°C. The supernatant was discarded, and the protein precipitates were washed with ice cold acetone before further centrifugation at 20,000 x g for 10 min at 4°C. The supernatant was discarded, and the protein pellets allowed to air dry before being solubilized for SDS-PAGE as above. For BN-PAGE analysis of pellet fractions, pellets were resuspended in 1% (w/v) digitonin buffer as previously described (Wittig et al., 2006; Lazarou et al., 2007) and subjected to shaking at 1500 rpm for 90 min at 4°C. Samples were centrifuged at 20,000 x g for 10 min at 4°C and BN-PAGE analysis and Western blotting were carried out as described above.

### 2.4 Sodium Carbonate Extraction for Mass Spectrometry

HEK293T, UQCRC1^KO^, and UQCR10^KO^ cells were cultured in DMEM for SILAC (Thermo Scientific, 88364), supplemented with 10% (v/v) dialyzed FCS (Thermo Scientific, 30067334), penicillin/streptomycin (Thermo Fisher Scientific, Scoresby, VIC, Australia), 50 μg ml^−1^ uridine (Sigma-Aldrich, North Ryde, NSW, Australia), 600 mg L^−1^ L-proline and, either regular “light” or “heavy” (^13^C_6_^15^N_2_ lysine and ^13^C_6_^15^N_4_-arginine) arginine and lysine (Silantes, 201604102 and 211604102) at the concentrations of 42 mg L^−1^ L-arginine and 146 mg L^−1^ L-lysine at 37°C and 5% CO_2_ for at least 5 passages. Cells were collected, washed in PBS and counted. Samples were prepared in triplicate with a label switch. Equal numbers of HEK293T cells (2 independent samples grown in heavy DMEM and one grown in light DMEM) and knockout cells (1 sample grown in heavy DMEM and 2 independent samples grown in light DMEM) were mixed in heavy/light HEK293T/knockout pairs and used for isolation of mitochondria as previously described (Stroud et al., 2016). SILAC-labelled mitochondria were pelleted at 12,000 x g for 5 min at 4°C before being resuspended in 100 mM Na_2_CO_3_, pH 9.5 or 11.5 and incubated on ice for 30 min. One fifth of the volume of each sample was aliquoted as the “Total” fraction. The remainder was centrifuged at 125,000 x g for 30 min at 4°C to generate “Pellet” and “Supernatant” fractions. Peptides were prepared using the S-Trap system (Hailemariam et al., 2018) as per manufacturer’s instructions. The total fraction was solubilized using 1X S-Trap solubilization buffer while pellet and supernatant fractions were prepared using 3X S-Trap solubilization buffer. The total fraction samples were processed using Micro S-Trap columns, while the pellet and supernatant fractions were processed using Mini S-Trap columns. Proteins were digested with trypsin (Thermo Fisher Scientific) at a 1:25 trypsin:protein ratio overnight at 37°C. Peptides were eluted from S-Trap columns according to manufacturer’s instructions, prior to drying in a CentriVap Benchtop Vacuum Concentrator (Labconco, Kansas City, MO) and reconstitution in 0.1% TFA and 2% ACN for analysis by Liquid chromatography (LC) - MS/MS on an Orbitrap Exploris 480 (Thermo Fisher Scientific) coupled with an Ultimate 3000 HPLC (Thermo Fisher Scientific) and NanoESI interface. The LC system was equipped with an Acclaim Pepmap nano-trap column (Dinoex-C18, 100 Å, 75 µm × 2 cm) and an Acclaim Pepmap RSLC analytical column (Dinoex-C18, 100 Å, 75 µm × 50 cm). The tryptic peptides were injected to the enrichment column at an isocratic flow of 5 µl/min of 2% v/v CH_3_CN containing 0.1% v/v formic acid for 5 min applied before the enrichment column was switched in-line with the analytical column. The eluents were 5% DMSO in 0.1% v/v formic acid (solvent A) and 5% DMSO in 100% v/v CH_3_CN and 0.1% v/v formic acid (solvent B). The flow gradient was 1) 0–6 min at 3% B, 2) 6–95 min, 3-22% B 3) 95–105 min 22–40% B 4) 105–110 min, 40–80% B 5) 110–115 min, 80–80% B 6) 115–117 min, 80–3% and equilibrated at 3% B for 10 min before the next sample injection. The mass spectrometer was operated in the data-dependent acquisition mode, whereby full MS1 spectra were acquired in a positive mode at 1,20,000 resolution. The “top speed” acquisition mode with 3 s cycle time on the most intense precursor ion was used, whereby ions with charge states of 2–7 were selected. MS/MS analyses were performed by 1.6 m/z isolation with the quadrupole, fragmented by HCD with collision energy of 30%. MS2 resolution was at 15,000. Dynamic exclusion was activated for 30 s. AGC target was set to standard with auto maximum injection mode. Dynamic exclusion was activated for 30 s. Raw files were processed using the MaxQuant software package (Version 1.6.17.0, RRID:SCR_014485) (Tyanova et al., 2016a), and searched against the UniProt human database, including unreviewed proteins, (193557 entries, March 2021). For this search, Arg10 and Lys8 were set as the heavy labels and Trypsin/P cleavage specificity (cleaves after lysine or arginine, even when proline is present) was used with a maximum of 2 missed cleavages. Oxidation of methionine and N-terminal acetylation were specified as variable modifications. Carbamidomethylation of cysteine was set as a fixed modification. The settings “LFQ,” “re-quant” and “Match between runs” were enabled with otherwise default settings.

### 2.5 Steady State Proteomics

SILAC labeled isolated mitochondria were prepared as described above, and peptides were prepared using Mini S-Trap columns (Hailemariam et al., 2018) as described above for analysis by LC-MS/MS on an Orbitrap Eclipse. The LC was carried out as described above. The mass spectrometer was operated in the data-dependent acquisition mode, whereby full MS1 spectra were acquired in a positive mode at 120,000 resolution. The “top speed” acquisition mode with 3 s cycle time on the most intense precursor ion was used, whereby ions with charge states of 2–7 were selected. MS/MS analyses were performed by 1.6 m/z isolation with the quadrupole, fragmented by HCD with normalized collision energy of 30%. MS2 resolution was at 15,000. Dynamic exclusion was activated for 30 s. AGC target was set to standard with auto maximum injection mode. Raw files were processed as described above, however without the “LFQ” option enabled.

### 2.6 Complexome Mass Spectrometry

SILAC labelled heavy UQCRC1^KO^/light HEK293T isolated mitochondria were prepared as described above and analyzed via BN-PAGE as described above. Detergent solubilized complexes were separated on a 4–16% acrylamide gel, run overnight. The blue native gel containing separated SILAC-labelled mitochondria was transferred into fixing solution (50% v/v methanol, 10% v/v acetic acid, 10 mM ammonium acetate) and incubated for 30 min, followed by a 30-minute incubation in Coomassie solution (0.025% (w/v) Coomassie, 10% v/v acetic acid). The gel was destained in 10% acetic acid for several hours until background was clear, then washed in water at least three times. The lane containing the SILAC-labelled mitochondria was excised and cut into 60 even slices. Each slice was further diced into smaller pieces and placed into individual wells of an Acroprep™ 30-40 μm PP/PE filtered microtiter plate containing 50 mM ammonium bicarbonate (ABC) for in-gel tryptic digest as previously described (Giese et al., 2021). Briefly, the ABC was removed by centrifugation before destaining. All centrifugation steps were performed at 1,500 x g. The destain and wash process was repeated 2–3 times as required. Gel peices were incubated for 60 min with gentle shaking in destain solution (60% methanol v/v, 50 mM ABC). Gel pieces were washed in 50% v/v acetonitrile (ACN), 50 mM ABC and incubated in 10 mM dithiothreitol, 50 mM ABC for 60 min at 56°C without shaking. Gel pieces were then incubated in 40 mM chloroacetamide, 50 mM ABC for 45 min before being washed twice with 50% v/v ACN, 50 mM ABC. Gel pieces were then allowed to air dry for 40–45 min without agitation, before digestion solution (10 ng/μl trypsin (Thermo Fisher Scientific), 10% v/v ACN, 0.01% (w/v) ProteaseMAX surfactant (Promega), 1 mM CaCl_2_, 50 mM ABC) was added, and topped up with 50 mM ABC, for overnight incubation at 37°C in a pre-humidified incubator. Digested peptides were eluted the next day into a fresh microtiter plate by centrifugation before adding additional elution buffer (30% [v/v] ACN, 3% [v/v] formic acid (FA)) to gel pieces before gentle shaking for a further 20 min. The second elution was collected by centrifugation and both elutions were pooled. Peptide solutions were dried using a CentriVap concentrator (Labconco) then reconstituted in 2% [v/v] ACN, 0.1% [v/v] TFA. Samples were desalted on stagetips containing 2x 14G plugs of 3M™ Empore™ SDB-XC extraction Disks (Sigma-Aldrich), which were pre-activated with 100% ACN and equilibrated with 2% [v/v] ACN, 0.1% [v/v] TFA before loading. Stagetips were washed with 2% [v/v] ACN, 0.1% [v/v] TFA and samples were eluted with 80% [v/v] ACN, 0.1% [v/v] TFA. All centrifugation steps were performed at 3,000 x g. Elutions were dried and reconstituted in 2% [v/v], 0.1% TFA [v/v] for LC-MS/MS analysis. The LC system was equipped with an Acclaim Pepmap nano-trap column (Dinoex-C18, 100 Å, 75 µm × 2 cm) and an Acclaim Pepmap RSLC analytical column (Dinoex-C18, 100 Å, 75 µm × 50 cm). The tryptic peptides were injected to the enrichment column at an isocratic flow of 5 µl/min of 2% v/v CH_3_CN containing 0.05% [v/v] TFA for 6 min applied before the enrichment column was switched in-line with the analytical column. The eluents were 5% DMSO in 0.1% [v/v] formic acid (solvent A) and 5% DMSO in 100% [v/v] CH_3_CN and 0.1% [v/v] formic acid (solvent B). The flow gradient was 1) 0-6min at 2% B, 2) 6–25 min, 2–23% B 3) 25–35 min 23–40% B 4) 35–40 min, 40–80% B 5) 40–42 min, 80–80% B 6) 42–42.1 min, 80–2% and equilibrated at 2% B for 10 min before the next sample injection. The QExactive plus mass spectrometer was operated in the data-dependent mode, whereby full MS1 spectra were acquired in positive mode, 70 000 resolution, AGC target of 3e^6^ and maximum IT time of 50 ms. Fifteen of the most intense peptide ions with charge states ≥2 and intensity threshold of 4e^4^ were isolated for MSMS. The isolation window was set at 1.2m/z and precursors fragmented using normalized collision energy of 30, 17 500 resolution, AGC target of 5e^4^ and maximum IT time of 50 ms. Dynamic exclusion was set to be 30sec. Raw files were processed using the MaxQuant platform (version 1.6.17.0) against the UniProt human canonical and isoforms database (42,360 entries, August 2020) using settings for the standard identification of light and heavy (Arg-10 and Lys-8) SILAC-labelled peptides. Oxidation of methionine and N-terminal acetylation were specified as variable modifications, and carbamidomethylation of cysteine was set as a fixed modification. Trypsin/P cleavage specificity was used with a maximum of 2 missed cleavages, and a search tolerance of 4.5 ppm was used for MS1 and 20 ppm for MS2 matching. False discovery rates (FDR) were determined through the target-decoy approach set to 1% for both peptides and proteins. The calculation of intensity-based absolute quantification (iBAQ) intensities was enabled with the Log fit function disabled.

### 2.7 Proteomics Data Analysis

The ProteinGroups.txt files generated by the SCE-MS search was imported into Perseus (version 1.6.15.0, RRID:SCR_015753) (Tyanova et al., 2016b). Normalized H/L Ratios and individual LFQ Heavy (H) and Light (L) intensities were Log_2_-transformed and grouped by cell line and fraction. Proteins were filtered for mitochondrial localization based on the MitoCarta3.0 dataset (Rath et al., 2021) added through matching by Uniprot ID. For comparing “Total” fractions and “Pellet” and “Supernatant” fractions prior to normalization, for each fraction, proteins were filtered for H/L ratio quantitation in at least two samples and a one-sample two-sided t-test was conducted with results expressed as a volcano plot. For normalized comparisons, Log_2_-transfrormed ratios of each replicate of the “Total” fraction was subtracted from the corresponding “Pellet” or “Supernatant” fraction. Proteins without quantified ratios in both “Total” and either “Pellet” or “Supernatant” fractions were excluded from comparisons. A one-sample two-sided t-test was conducted, and results expressed as a volcano plot. For comparisons between “Supernatant” and “Pellet” fractions in the same cell line, proteins were filtered for those quantified in at least two third of samples for at least two of pH 9.5 “Pellet”, pH 9.5 “Supernatant”, pH 11.5 “Pellet” or pH 11.5 “Supernatant”. The number of transmembrane domains for each protein were added using the Uniprot database (The Uniprot Consortium, 2021). Missing values were imputed based on the normal distribution with a width of 0.3 and a down-shift of 1.8, and a two-sample t-test was performed between “Pellet” and “Supernatant” groups with the results displayed as a volcano plot. For cluster analysis, each fraction was averaged and Z-scored. Hierarchical clustering was then performed with default settings. Clusters were defined in HEK293T samples using the define row cluster tool. Samples were then un-Z-scored and principal component analysis plots generated. For transmembrane domain prediction, the FASTA sequences of proteins in cluster 1 were analyzed using HMMTOP (Bioinformatics Toolkit, RRID:SCR_010277) (Tusnády and Simon, 2001), TMHMM (RRID:SCR_014935) (Krogh et al., 2001), TMPred (Hofmann, 1993) and Phobius (RRID:SCR_015643) (Käll et al., 2004).

For the steady-state experiment, the ProteinGroups.txt file was imported into Perseus. Normalized H/L ratios were Log_2_-transformed and grouped by cell line. Proteins were filtered for mitochondrial localization based on Mitocarta3.0 and ratios were normalized by subtracting the median of each column. A one-sample two-sided t-test was conducted, and result expressed as a volcano plot and topographical heatmap as previously described (Stroud et al., 2016) with -2 and 2 as minimum and maximum values respectively.

For complexome analysis, heavy and light iBAQ intensities were imported into Perseus (version 1.6.15.0), filtered and annotated for mitochondrial proteins listed in MitoCarta 3.0 (Rath et al., 2021), then imported into NOVA (version 0.8.0.0) for complexome analysis and visualisation. Mass scale calibration was performed by exponential interpolation in NOVA by inputting apparent protein masses selected from Supplementary Table S5 from (Maclean et al., 2021). Selected proteins of interest underwent hierarchical clustering using default settings, with optimised leaf ordering, average linkage, and a Person Correlation Distance function, without normalisation. Exported heatmaps with iBAQ values were normalised in Excel, by row average for heatmap generation, and by maximum intensity for profile plot generation. Figures were generated in GraphPad Prism 9 (RRID:SCR_002798).

### 2.8 Oxygen Consumption Measurements

Mitochondrial oxygen consumption rates were measured in live cells using a Seahorse Bioscience XFe-96 Analyzer (Agilent Mulgrave, VIC, Australia). Briefly, 25,000 cells were plated per well in culture plates treated with 50 μg/ml poly-d-lysine (Sigma-Aldrich). For each assay cycle, there were 3 measurements of 2 min followed by mixing, 2 min wait, and 3 min measure. The following inhibitor concentrations were used: 1 μm oligomycin, 1 μm carbonyl cyanide 4-(trifluoromethoxy)phenylhydrazone (FCCP), 0.5 μm rotenone and 0.5 μm antimycin A. Data were normalized using the Pierce Protein Assay Kit (Thermo Fisher Scientific) and analyzed using Wave (version 2.6.0, Agilent, RRID:SCR_014526) and Prism (version 8.0.2, GraphPad, RRID:SCR_005375) software packages.

## 3 Results

**FIGURE 1:**
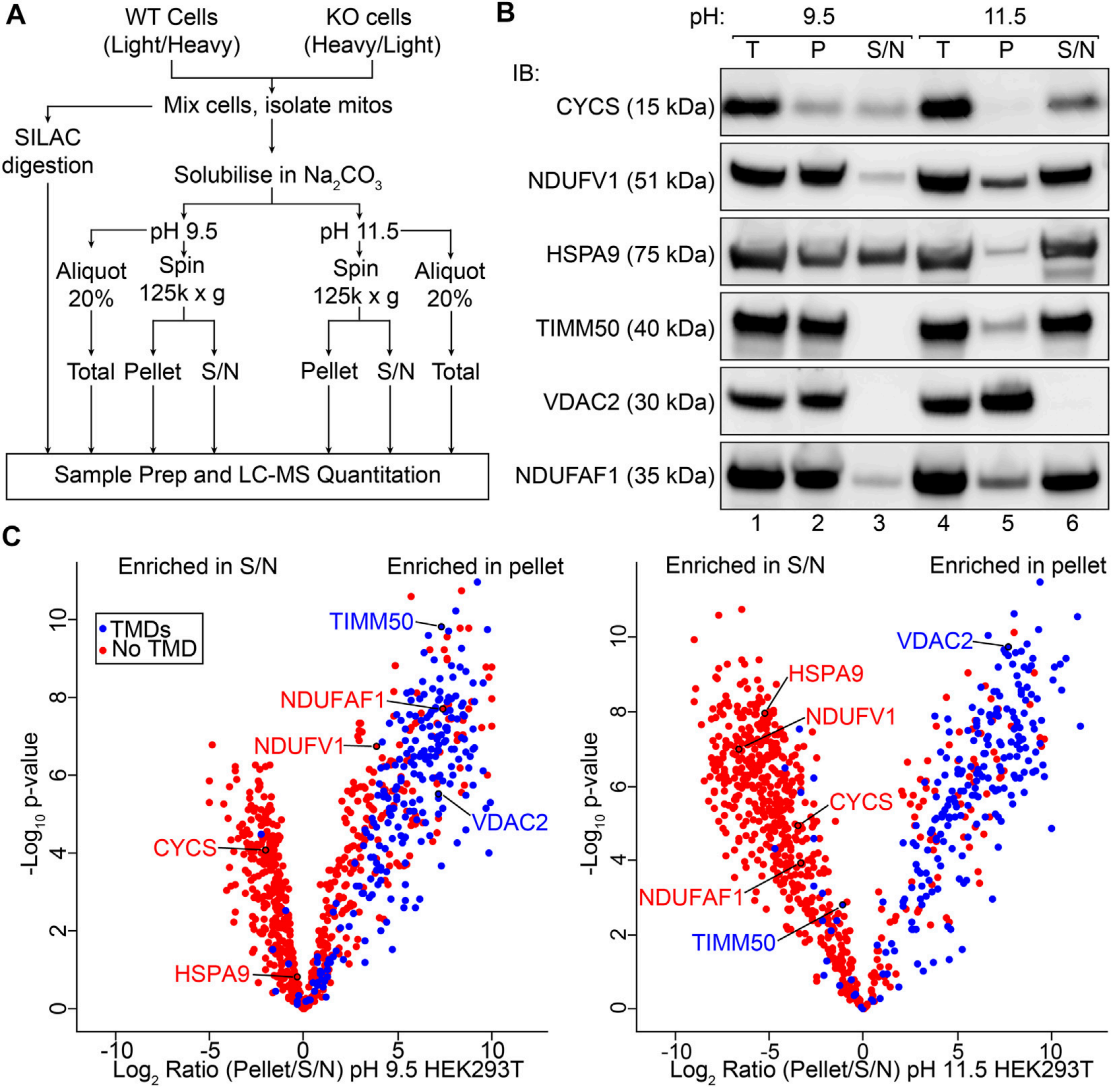
Sodium carbonate extraction MS to assess membrane association on a mitochondrial proteome wide scale. **(A)** Protocol for SILAC coupled sodium carbonate extraction experiment. SILAC heavy and light labeled WT and KO cells are mixed and mitochondria isolated from the resultant pool of cells. Pooled mitochondria are then used directly for quantitative mass spectrometry or for sodium carbonate extraction (Na_2_CO_3_) MS. Total, Pellet and Supernatant (S/N) fractions generated by carbonate extraction and analyzed by LC-MS. **(B)** Mitochondria isolated from the HEK293T cell line were subjected to sodium carbonate extraction and total (T), pellet (P) and supernatant (S/N) fractions were analyzed by SDS-PAGE and immunoblotting (IB) with indicated antibodies. **(C)** Volcano plots depicting relative abundance of mitochondrial proteins in the pellet and supernatant fractions after sodium carbonate extraction at pH 9.5 and 11.5.

To assess the membrane association of the mitochondrial proteome in an unbiased manner, we adapted a sodium carbonate extraction protocol for use with quantitative proteomics workflows (Figure 1A). This included the use of stable isotope labelling using amino acids in cell culture (SILAC) to allow the impact of perturbations (such as CRISPR/Cas9 knockout of a protein of interest) to be quantitatively assessed, as discussed in subsequent results sections. In order to validate our protocol, we first analyzed sodium carbonate extraction data for only control HEK293T mitochondria, separated from the dataset *in silico* and treated in a similar way as would be done in a label free quantification (LFQ; see *Materials and Methods* for more detail)*.* SDS-PAGE and immunoblotting using antibodies against mitochondrial proteins with varied localizations and membrane integration was also performed to establish baseline profiles for proteins with known behavior (Figure 1B). At pH 11.5, outer membrane channel VDAC2 and peripheral IMM electron carrier cytochrome *c* (CYCS) are found exclusively in the pellet and supernatant fractions respectively as expected (Ma et al., 2014;Letts et al., 2016). Complex I N-module/matrix arm subunit NDUFV1 and assembly factor NDUFAF1, both peripherally associated with the membrane (Stroud et al., 2016), along with import translocase components TIMM50 and mt-HSP70 are found in both fractions at pH 11.5. At pH 9.5, a reduction in carbonate extraction is observed for each protein, with only cytochrome *c* and mt-HSP70 found in equal proportions in both the pellet and supernatant fractions, possibly due to their localization in both soluble and peripherally membrane associated pools (Kang et al., 2018;Timón-Gómez et al., 2018). Using SCE-MS we were able to quantify the relative abundance of 844 mitochondrial proteins (Rath et al., 2021) from control HEK293T cells at both pH 9.5 and 11.5 (Figure 1C; Supplementary Table S1). This represents 74% of the known mitochondrial proteome. Under both extraction conditions the majority of proteins with transmembrane domains annotated in UniProt (The Uniprot Consortium, 2021) were detected at greater abundance in the pellet fraction. Proteins without annotated TMDs were found most enriched in the supernatant extracted at pH 11.5, but were more evenly distributed between supernatant and pellet fractions following extraction at the lower pH, as expected.

**FIGURE 2:**
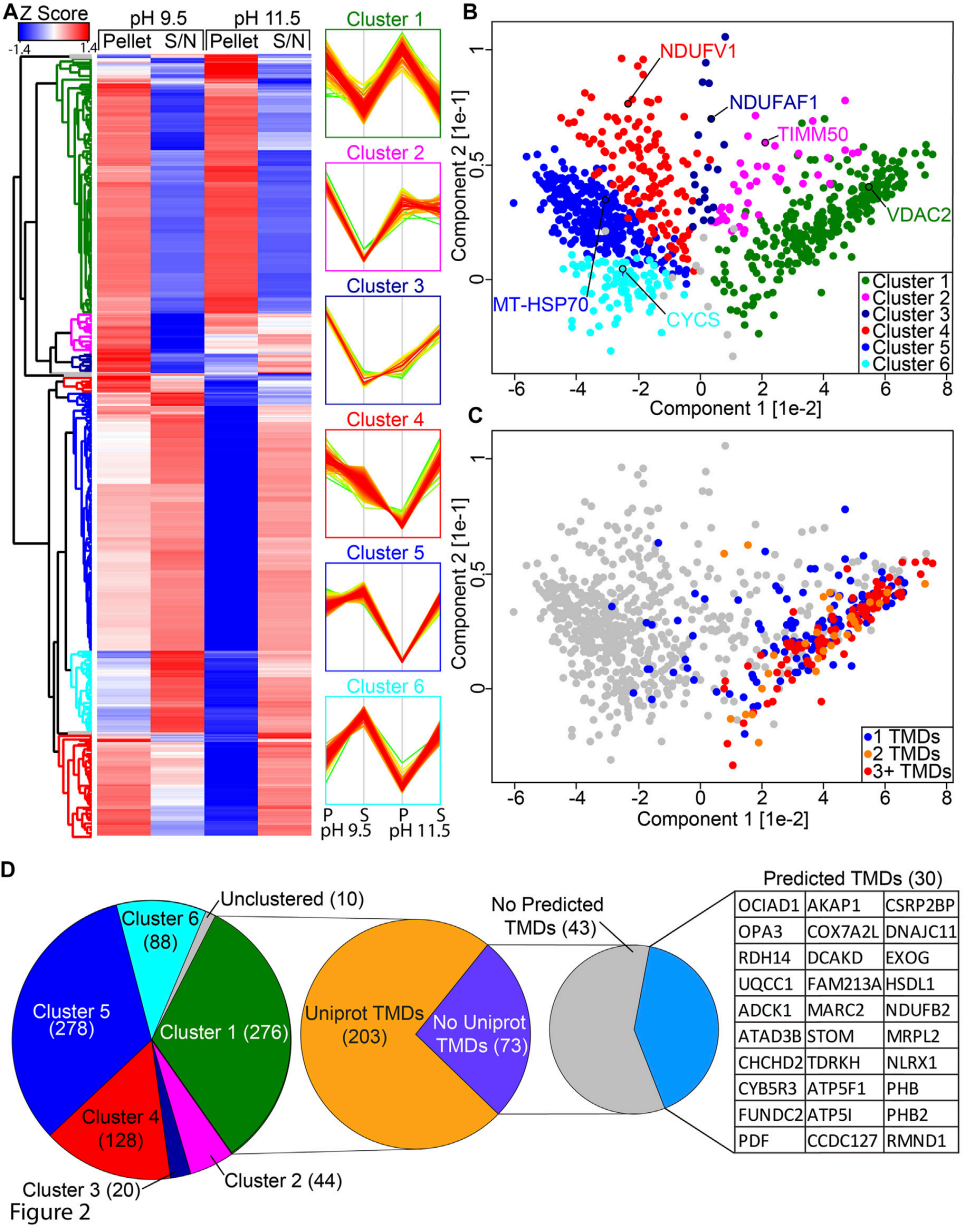
Classification of protein clusters. **(A)** HEK293T mitochondrial proteome SCE-MS data was Z-scored and hierarchical clustering was performed. Results are expressed in a heatmap and the profiles of each cluster are shown. The *x*-axis on the profiles indicates relative abundance, where higher is equal to more abundant. Coloring reflects distance to the mean of the profile, where red is closest, green is most distant. **(B)** Principal component analysis of HEK293T supernatant and pellet fractions at pH 9.5 and 11.5, colored according to the clusters identified in **(A)**. **(C)** as for B but with coloring according to the number of transmembrane domains. **(D)** Breakdown of Cluster 1 showing number of proteins with TMDs annotated in Uniprot and predicted by at least two single TM prediction algorithms (TMPred, HMMTOP, TMHMM or Phobius).

In order to classify the mitochondrial proteome based on extraction properties, we performed hierarchical clustering on our SCE-MS data (Figure 2A; Supplementary Table S2) and used this to define six distinct clusters that could be readily visualized using principal component analysis (PCA; Figure 2B; Supplementary Table S2). Cluster 1 contains 276 proteins that are resistant to membrane extraction at both pH 9.5 and 11.5. This includes hydrophobic proteins such as mitochondrial transporter family SLC25A members and all detected mtDNA encoded proteins. Cluster 2 contains 44 proteins that are resistant to extraction at pH 9.5 but are partially susceptible to extraction at pH 11.5. Cluster 3 contains 20 proteins that are resistant to extraction at pH 9.5 but very susceptible at pH 11.5. Cluster 4 contains 128 proteins that are moderately resistant to extraction at pH 9.5 but highly susceptible at pH 11.5, including 21 subunits of the small subunits of the mitoribosome, 16 of the large subunit and 6 out of the 9 detected subunits of the N-module of complex I. Cluster 5 contains 278 proteins that are partially resistant to extraction at pH 9.5 but very susceptible at pH 11.5. Cluster 6 contains 88 proteins that are susceptible to extraction at both pH 9.5 and 11.5. A total of 10 proteins quantified in this analysis did not segregate into a cluster.

To further validate our dataset, we cross-referenced the number of predicted TMDs present in proteins found within each cluster. Predictions were drawn from UniProt (The Uniprot Consortium, 2021), which annotates TMDs automatically based on the TMD being identified by both TMHMM (Krogh et al., 2001) and Phobius (Käll et al., 2004) algorithms with substantial overlap. Nearly every protein with two or more annotated TMDs was found in cluster 1 (Figure 2C), along with the majority of single TMD proteins. The remaining proteins with single TMDs are distributed across the other clusters, with a bias toward clusters 2 and 4. Of the 276 proteins in cluster 1, 73 had no TMD annotated in Uniprot (Figure 2D). Using a number of additional bioinformatics tools for predicting TMDs, including TMPred (Hofmann, 1993) and HMMTOP (Bioinformatics Toolkit, RRID:SCR_010277) (Tusnády and Simon, 2001), as well as TMHMM (RRID:SCR_014935) (Krogh et al., 2001) and Phobius (RRID:SCR_015643) (Käll et al., 2004) without the overlap constraint used by UniProt, we identified an additional 30 proteins that had TMDs identified by at least two algorithms. A number of these proteins have been experimentally determined to be genuine membrane proteins, including complex I subunit NDUFB2 through high resolution structural studies (Zhu et al., 2016) recently identified complex III assembly factor OCIAD1 through classical carbonate extraction studies (Le Vasseur et al., 2021) and pro-survival protein FUNDC2 through mutation and localization analysis (Ma et al., 2019).

**FIGURE 3:**
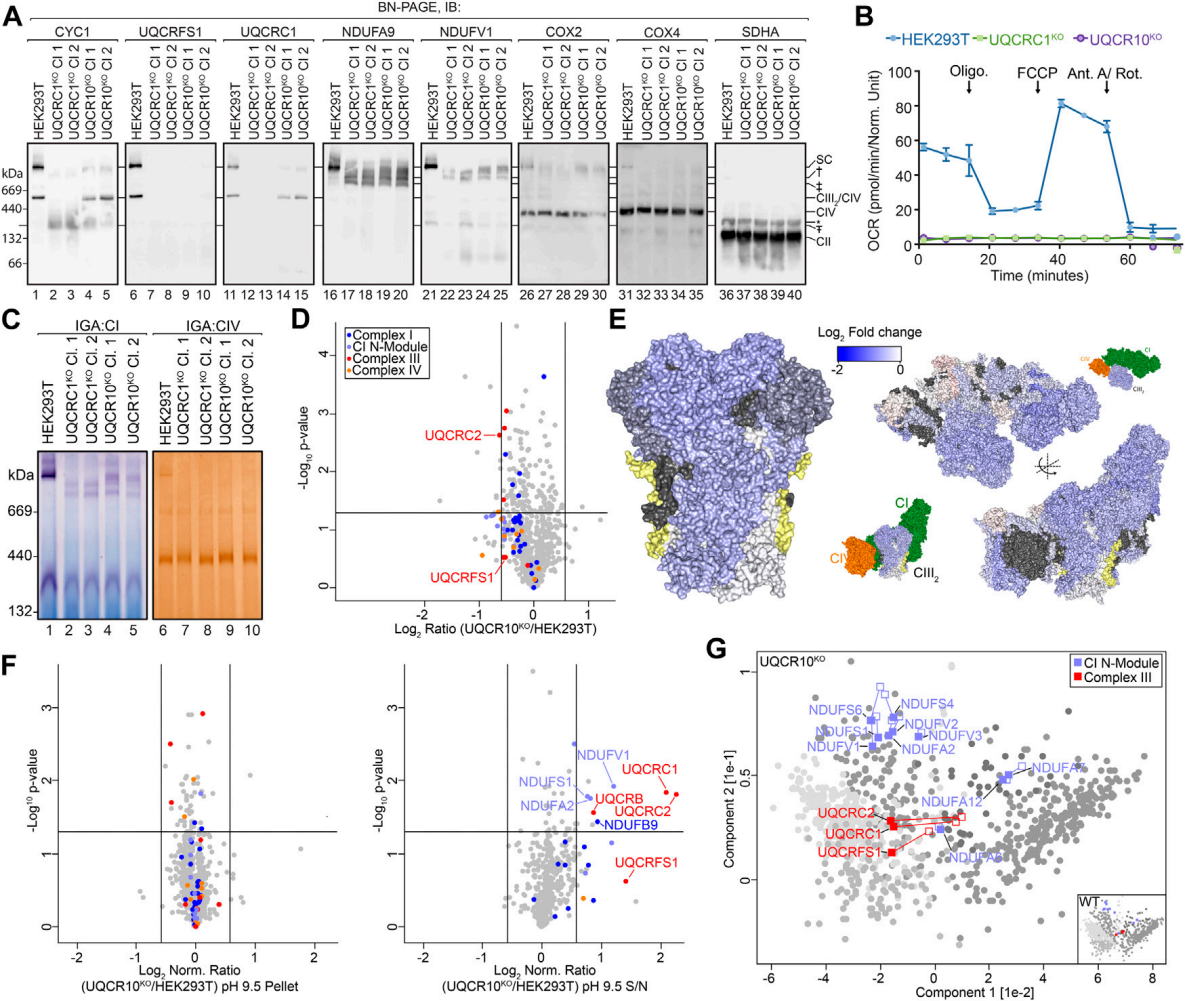
Loss of UQCR10 leads to destabilization and accumulation of a soluble core-module. **(A)** Mitochondria isolated from indicated cell lines were solubilized in 1% digitonin and analyzed by BN-PAGE and immunoblotting with the indicated antibodies. Ŧ, † and ‡ represent sub-assemblies discussed in text. *non-specific complex. **(B)** Oxygen consumption rates of HEK293T, UQCRC1^KO^ and UQCR10^KO^ cell lines. Oligo, oligomycin; FCCP, carbonyl cyanide 4-(trifluoromethoxy)phenylhydrazone; Ant A, antimycin A; Rot, rotenone. **(C)** Mitochondria isolated from indicated cell lines were analyzed using BN-PAGE and the activity of complex I and IV determined through in-gel assays. **(D)** Volcano plot depicting relative abundances of mitochondrial proteins in UQCR10^KO^ compared to HEK293T. **(E)** Topographical heatmap showing changes in abundance of CIII and respirasome subunits in UQCR10^KO^ using the CIII (PDB 5XTE) and respirasome (PDB 5XTH) structures. UQCR10 shown in yellow. **(F)** Volcano plots depicting relative abundance of proteins in pH 9.5 carbonate extraction pellet and supernatant fractions in UQCR10^KO^ compared to HEK293T mitochondria following normalization against the total fraction. Data prior to normalization and pH 11.5 data can be found in Supplementary Figures S1, S2. **(G)** Principal component analysis showing overall membrane association profiles in UQCR10^KO^ relative to HEK293T mitochondria. Complex I N-module and CIII subunits are labeled, with their position in control data shown as empty squares and position in knockout data as filled squares. Clusters defined in Figure 2A are colored in grey.

**FIGURE 4:**
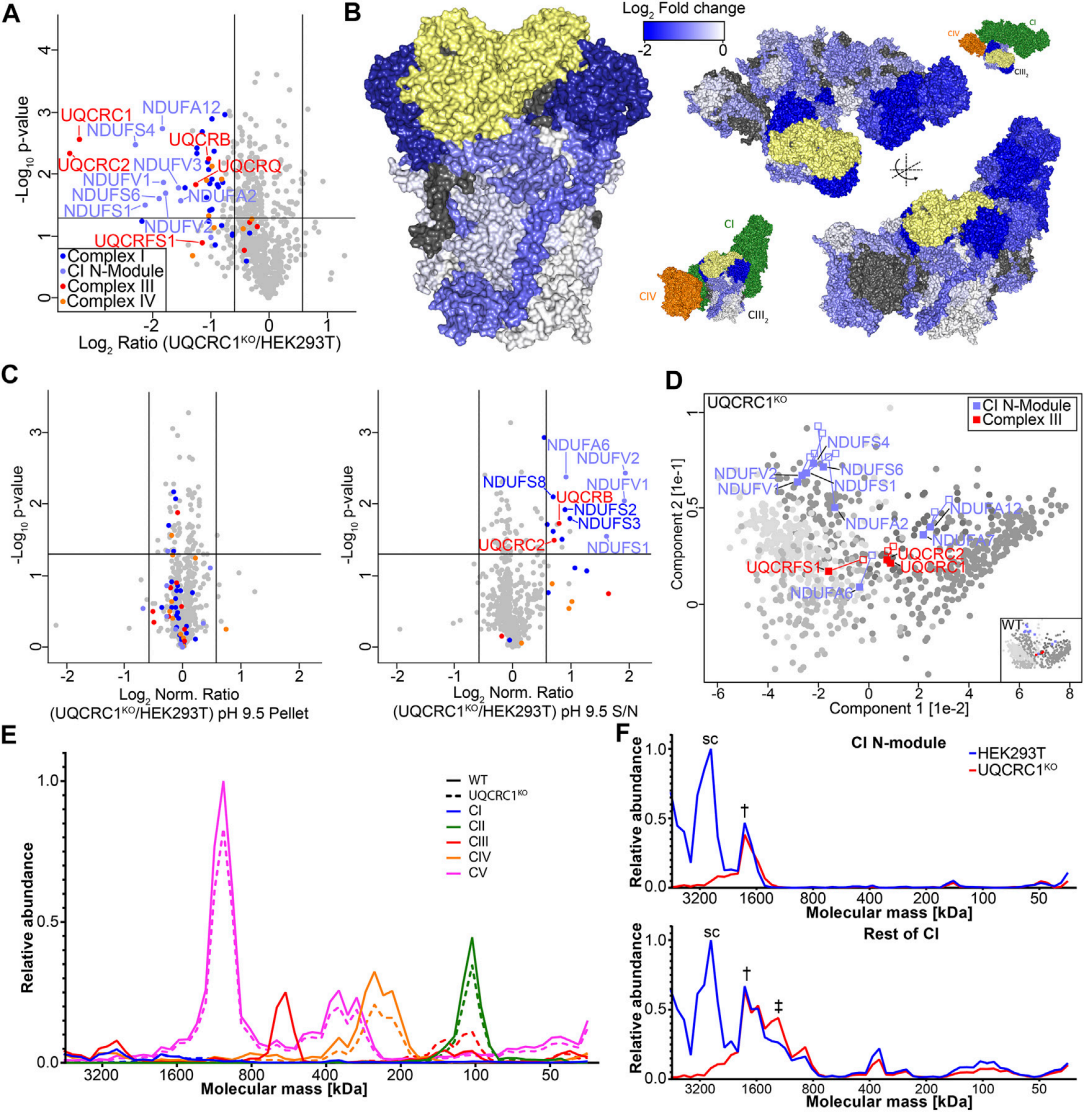
Loss of UQCRC1 destabilizes the N-module of complex I. **(A)** Volcano plot depicting relative abundances of mitochondrial proteins in UQCRC1^KO^ compared to HEK293T. **(B)** Topographical heatmap showing changes in abundance of CIII and respirasome subunits in UQCRC1^KO^ using the CIII (PDB 5XTE) and respirasome (PDB 5XTH) structures. UQCRC1 shown in yellow **(C)** Volcano plots depicting relative abundance of proteins in pH 9.5 carbonate extraction pellet and supernatant fractions in UQCR10^KO^ compared to HEK293T mitochondria following normalization against the total fraction. **(D)** Principal component analysis showing overall membrane association profiles in UQCRC1^KO^ relative to control mitochondria. Complex I N-module and CIII subunits are labeled, with their position in control data shown as empty squares and position in knockout data as filled squares. Clusters defined in Figure 2A are colored in grey. **(E)** Complexome profile of mitochondrial respiratory complexes in UQCRC1^KO^ and HEK293T mitochondria. Graphs plot relative intensities of peptides averaged across all subunits of each complex with the maximum set as 1.0. Molecular mass calculated as described in *Materials and Methods*. **(F)** Complexome profile of CI subunits, separated into N-module and the rest of the complex, as in **(E)**. The symbols used to indicate peaks represent the equivalent complex in Figure 3A.

Next, we wanted to demonstrate the usefulness of SCE-MS in identifying changes in membrane integration following a perturbation. We used the CRISPR/Cas9 system to generate knockout cell lines where the expression of complex III subunits UQCRC1 and UQCR10 was disrupted. Two individual clones for each knockout were validated by sequencing of targeted alleles (see *Materials and Methods*). To determine changes in OXPHOS complex assembly, we analyzed mitochondria from two clones of each knockout using Blue Native (BN) PAGE and immunoblotting (Figure 3A). Mitochondria from both knockouts retained a ∼150 kDa subassembly containing CYC1 that was more abundant in the UQCRC1^KO^ than UQCR10^KO^ (Figure 3A, compare lanes 2–5 with 1; subassembly indicated by Ŧ). Cells lacking UQCR10 retained a CIII_2_-like assembly containing CYC1 and UQCRC1 (compare lanes 4–5 with 1). No signal for complex III subunit UQCRFS1 was observed in either UQCRC1^KO^ or UQCR10^KO^ clones (compare lanes 7–10 with 6), indicating that UQCRFS1 is not assembled into CIII in either knockout cell line in agreement with existing assembly models that describe incorporation of that subunit to be a late stage assembly event (Fernandez-Vizarra and Zeviani, 2018), and despite UQCRFS1 levels being only slightly reduced in UQCR10^KO^ (see Figure 3D and Figure 4A; UQCRFS1 is decreased by a fold-change of ∼2 in UQCRC1^KO^ ). Interestingly, the CIII_2_-like complex in UQCR10^KO^ appeared to migrate slightly faster than wildtype CIII_2_ (Figure 3A), leading us to speculate that it could represent the pre-CIII_2_ intermediate (Fernandez-Vizarra and Zeviani, 2018). In the absence of peripheral core-module subunit UQCRC1, no supercomplex assemblies were observed, with CI migrating in two major sub-assemblies (compare lanes 22–23 with 21; assemblies indicated by † and ‡). In the absence of single-spanning membrane subunit UQCR10, similar CI sub-assemblies were observed, in addition to a supercomplex-like structure, albeit at a lower abundance than in control cells (compare lanes 24–25 with 21). In both UQCRC1^KO^ and UQCR10^KO^, assembly of holo CIV appears to be unperturbed, however higher order CIV assemblies are mostly absent, with the exception of a low abundance complex migrating at a similar molecular weight to the supercomplex in UQCR10^KO^. Given the reported role of CIII in CI and CIV assembly and function (Protasoni et al., 2020) and the unexpected impact of the loss of UQCRC1 and UQCR10 on CI seen in Figure 3A, we wanted to determine if CI and CIV activity was retained in our knockouts. Surprisingly, despite the loss of catalytic complex III subunit UQCRFS1 from all CIII-containing complexes and sub-assemblies (Figure 3A) and thus the loss of overall respiration via OXPHOS (Figure 3B), partial complex I in-gel activity was observed in the remnant supercomplex-like structure in UQCR10^KO^ (Figure 3C, compare lanes 4–5 with 1) and in the lower molecular weight assemblies seen in both knockouts (Figure 3C). Despite the low abundance band seen in immunoblotting, no Complex IV activity was observed in the supercomplex-like structure found in UQCR10^KO^, with the only activity found holo CIV (Figure 3C). Given the reproducible phenotype between multiple independent sequence-verified clones complementation experiments were not performed. Clones 1 of UQCRC1^KO^ and UQCR10^KO^ were used for all subsequent experiments.

We first focused our SCE-MS analysis on UQCR10 knockout mitochondria. Assembly of the dimeric pre-CIII_2_ occurs with the addition of membrane subunits UQCR10, CYC1 and UQCRH and peripherally associated core-module subunits UQCRC1 and UQCRC2 (Stephan and Ott, 2020). We reasoned that loss of UQCR10 may result in accumulation of a soluble core-module observable through SCE-MS, demonstrating how the technique can provide additional insights into CIII assembly. First we assessed the baseline steady-state levels of proteins in UQCR10^KO^ and controls using quantitative mass spectrometry of isolated mitochondria, as baseline data would be critical to interpretating SCE-MS data since some proteins may have an overall reduced cellular abundance due to perturbed assembly. Knockout and control HEK293T cells were subjected to SILAC labeling using light or heavy (^13^C_6_^15^N_2_ lysine and ^13^C_6_^15^N_4_-arginine) amino acids, and the relative abundance of mitochondrial proteins in the knockouts compared to HEK293T was determined. In total, we quantified relative changes in abundance of 749 mitochondrial proteins (Supplementary Table S3). While UQCR10 itself was not detected in UQCR10^KO^ mitochondria (Supplementary Table S3), OXPHOS subunits including most CIII subunits were only minimally reduced in abundance, with only UQCRC2 reaching our significance threshold of 1.5 fold change (Figure 3D). The non-specific trend in reduced abundance of CIII and supercomplex subunits is also evident using topographical heatmaps (Stroud et al., 2016) (Figure 3E) where the fold change for individual proteins observed in steady state proteomics is mapped to the cryo-electron microscopy structure of the human CI/CIII_2_/CIV supercomplex (Gu et al., 2016).

We next turned to SCE-MS, which was performed as outlined in Figure 1A using SILAC labelled UQCR10^KO^ and control cell lines (Supplementary Figure S1, Supplementary Table S4). To account for the overall decreased steady-state abundance of some proteins seen in Figure 3D we also measured proteome changes in a “Total” fraction containing sodium carbonate solubilized material prior to ultracentrifugation (Supplementary Figure S1, left panels), using this data to normalize pellet and supernatant SCE-MS results (Figure 3F shows normalized data at pH 9.5. Normalized data for pH 11.5 can be found in Supplementary Figure S2, and raw ratios in Supplementary Figure S1). Following carbonate extraction at pH 9.5, the normalized pellet fraction had no significant changes in the levels of OXPHOS subunits. However in contrast, CIII subunits UQCRC1 and UQCRC2 were found to be relatively more abundant in supernatant in UQCRC10^KO^. Similar results were observed following extraction at pH 11.5, however under these conditions the effect was reversed, with UQCRC1 and UQCRC2 being less abundant in the pellet fraction of UQCRC10^KO^ (Supplementary Figure S2B). To more easily visualize changes in membrane association of CIII subunits we compared the proteome wide changes to membrane association across both extraction conditions, assigning protein solubility in the knockout to the previously identified clusters (Figure 2B). While in the knockouts most proteins fell into the same clusters as in control cells, the clustering of core-module subunits UQCRC1 and UQCRC2 changed from clusters 2 to 5 in the knockout (Figure 3G, compare filled to empty squares) indicating altered membrane association. Also notable is UQCRFS1, the last assembled subunit (Fernandez-Vizarra and Zeviani, 2018), which moved from cluster 2 to 5. Cluster 5 contains proteins that are partially resistant to extraction at pH 9.5 but very susceptible at pH 11.5 and predominantly includes soluble matrix proteins such as those found in the mitoribosome and various tRNA ligases, along with proteins having mixed peripheral and soluble localization such as mt-HSP70 (Kang et al., 2018; Timón-Gómez et al., 2018) (Supplementary Table S2). These data, taken together with our BN-PAGE analysis that shows UQCRC1 remains associated with CIII_2_ in the UQCR10^KO^ under native-electrophoresis conditions (Figure 3A, compare lanes 14–15 with 11), suggest that the interaction between core module subunits UQCRC1 and UQCRC2 and the nascent CIII membrane assembly is destabilized in the absence of UQCR10, evidenced by the increased resistance of UQCRC1 and UQCRC2 to extraction at pH 9.5.

A concern we had over the validity of our SCE-MS data is the impact of the carbonate extraction procedure on protein complex stability. Our knockouts harbor sub-assemblies with different protein composition to that found in control cells. Should the presence of carbonate lead to selective partial breakdown of these complexes but not the comparable control complex, this would potentially lead to artefactual results caused by altered extractability of these breakdown sub-complexes. An example sub-assembly would be the CIII_2_-like complex lacking UQCRFS1 and UQCR10 that we suspect may represent pre-CIII_2_ (Figure 3A, compare lanes 4–5 with 1), as in the above experiments we are directly comparing the extractability of UQCRC1 and UQCRC2 from this and wildtype holo-CIII_2_ (along with other assemblies). To address this, we analyzed membrane fractions from our control and knockout cells extracted at both pH 9.5 and 11.5 using BN-PAGE and immunoblotting (Supplementary Figure S3). While the relative abundance of some sub-assemblies changed under different conditions as expected based on our SCE-MS data, their migration on BN-PAGE did not. We also did not observe any sub-complexes unique to either carbonate extraction condition. Taken together, we conclude that the presence of sodium carbonate does not alter the composition of the complexes and sub-assemblies being discussed in this study.

We next turned our attention to the UQCRC1 knockout. To obtain a baseline of proteome changes in the knockout, we performed steady state quantitative mass-spectrometry on isolated mitochondria, as described above (Supplementary Table S3). Loss of UQCRC1 led to a significant decrease in the levels of subunits present in CI, CIII and CIV, particularly the subunits of the core module of CIII, UQCRC1 and UQCRC2, and of the N-module of Complex I (Figure 4A). We again used topographical heatmaps to interpret the data in the context of the CI/CIII_2_/IV supercomplex (Figure 4B) (Gu et al., 2016), which clearly visualized the greatest impact of UQCRC1 loss to be on the core module of CIII and the N-module of Complex I. While this is consistent with recent reports that dysfunctional CIII assembly prevents addition of the N-module to Complex I (Protasoni et al., 2020), the molecular underpinnings of this phenomenon are not fully understood. To investigate this further, we performed SCE-MS on UQCRC1^KO^ as described above. In mitochondria from this knockout we observed a significant increase in the solubility of several CI subunits, with the greatest increase seen in N-module subunits (Figure 4C, right panel). Also moderately increased in solubility were the CIII subunits UQCRC2 and UQCRB. As done for UQCR10^KO^, we used a PCA-based approach incorporating both extraction conditions to better understand the implication of proteome solubility changes seen in the UQCRC1^KO^. While most proteins quantified in UQCRC1^KO^ could be assigned to the same clusters as in control cells (Figure 2B), the clustering of Complex I N-module subunits changed (Figure 4D, compare filled to empty squares) indicating altered membrane association. In the control HEK293T dataset, whereas most N-module subunits appeared at the intersect of cluster 3 and 4, these proteins migrated toward cluster 5 in UQCRC1^KO^ (Figure 4D). Complex III subunits were largely unchanged, with the exception of UQCRFS1, which like in UQCR10^KO^ moved from cluster 2 to 5. Similar trends in respect to N-module solubility were observed, though with less severity, for UQCR10^KO^ (Figure 3G). Despite N-module subunits being increasingly soluble in UQCRC1^KO^ and given the results of Protasoni and colleagues (2020) we were surprised by the relatively large amount of catalytic N-module subunit NDUFV1 still migrating with other Complex I subunits in UQCRC1^KO^ (Figure 3A), and moreover, CI-linked NADH dehydrogenase activity, an enzymatic reaction performed by the N-module, retained in UQCRC1^KO^ (Figure 3C). Interestingly, Protasoni and colleagues (2020) also observed low amounts of Complex I activity in their model of CIII dysfunction, however complexome profiling, a technique where lanes from BN-PAGE gels are excised into ∼60 slices and the protein distribution obtained by MS analysis, showed that the majority of holo-Complex I lacked associated N-module subunits. To better understand our results, we performed complexome profiling on the UQCRC1^KO^ (Figure 4E; Supplementary Figure S4; Supplementary Table S5) which accurately recapitulated observations we made using traditional BN-PAGE and immunoblotting (Figure 3A). A closer inspection of the dataset revealed multiple high molecular weight Complex I sub-assemblies in UQCRC1^KO^, including a dominant assembly containing all Complex I subunits, and at least two assemblies lacking the N-module (Figure 4F, † indicates assembly containing N-module, ‡ the assembly lacking N-module observed in Figure 3A). Taken together with our SCE-MS and in-gel activity data, we conclude that while the severe structural defect in CIII assembly found in UQCRC1^KO^ leads to the association between the N-module and the Complex I membrane assembly being destabilized, the N-module still retains an ability to associate with the complex. We suspect the differences in N-module association between our study and Protasoni et al. (2020) can be attributed to cell type and other confounders (see *Discussion*), and while our data broadly supports their conclusions it raises additional questions as to the molecular nature of Complex I N-module assembly.

## 4 Discussion

Sodium carbonate extraction is technique commonly used to identify integral membrane proteins. The technique has been adapted for use in both immunoblotting and radiolabeled *in vitro* import based assays (Stojanovski et al., 2007) and in addition to being useful in the characterization of newly identified proteins, is commonly used to gain mechanistic insights into membrane protein complex biogenesis through the use of mutant and gene-edited cell lines where the assembly process is blocked at specific steps. The use of multiple pH extraction conditions and control proteins with known localization additionally allows proteins with peripheral membrane association to be assessed (Lytovchenko et al., 2014). Here we describe a technique that combines sodium carbonate extraction with quantitative proteomics applicable for routine use in the study of mitochondrial complex assembly. Sodium carbonate extraction has previously been coupled with mass-spectrometry (Vögtle et al., 2017) however in that study the technique was combined with several other biochemical fractionation protocols to develop a steady state map of the *Saccharomyces cerevisiae* mitochondrial proteome. Vögtle and colleagues utilized SILAC multiplexing to compare crude and highly pure yeast mitochondria, allowing novel mitochondrial proteins to be confidently identified (Vögtle et al., 2017), but making their protocol not applicable to routine comparative analysis. Using SCE-MS we profiled the membrane association of 844 mitochondrial proteins in the commonly used human HEK293T cell line, roughly 75% of the human mitochondrial proteome (Rath et al., 2021). We applied bioinformatic tools to this dataset in order to cluster proteins based on their membrane association profiles, identifying 6 well defined clusters with decreasing membrane association. Clusters were validated against proteins with known membrane association and readily separate proteins with genuine membrane integration via TMDs (Cluster 1) such the polytopic SLC25A family of solute carriers found in the IMM (Palmieri, 2013) from truly soluble proteins (Cluster 6). One interesting outcome of this analysis is the large proportion of proteins that behave as peripherally associated membrane proteins. We identified four different clusters, accounting for almost 60% of proteins quantified, with varying levels of extraction at both pH 11.5 but partial resistance to extraction at pH 9.5, suggesting at least some peripheral association with the membrane. Our dataset, available in Supplementary Table S2, can be used as a general reference for the membrane association of a protein of interest in HEK293T cells.

One caveat of our dataset that could explain the profiles seen for some proteins is the tendencies for large soluble complexes such as the mitoribosome (subunits of which are predominantly found in Cluster 4) to sediment at the high centrifugation speeds used in our assay (Aibara et al., 2020). This highlights the need for biology and the nature of co-clustering proteins to be considered when interpreting SCE-MS data. For example, the mitoribosome is known to associate with the IMM translocase OXA1 to facilitate co-translational membrane insertion (Itoh et al., 2021) while Cluster 4 also contains subunits found in the membrane associated matrix arm of complex I. Together these lines of evidence suggest the presence of mitoribosome proteins in this cluster may reflect at least some degree of membrane association. To further test our clusters for biological significance we assessed the number of TMDs predicted for each protein. As expected, the majority of proteins with predicted TMDs were resistant to extraction and as such clustered together in Cluster 1. Through bioinformatic analysis of Cluster 1 proteins, we identified 30 proteins with likely TMDs that are not annotated on the Uniprot database. The presence of the other 43 proteins lacking annotated TMDs within Cluster 1 suggests that these proteins may contain transmembrane beta barrels or may be associated with the membrane through post-translational modifications, such as glycosylphosphatidylinositol (GPI) anchors or covalent lipid modifications. While proteins with two or more TMDs are almost exclusively found within Cluster 1, a number of single TMD proteins were found in all other clusters, including two proteins with single TMDs in Cluster 6, which is made up of mostly soluble proteins susceptible to carbonate extraction. One of these, the serine protease HTRA2, contains an N-terminal transmembrane domain that is inserted into the IMM during import, the protein is subsequently cleaved, releasing the soluble domain into the IMS (Challa et al., 2007).

As a test case for SCE-MS as a tool to complement techniques such as BN-PAGE in understanding the assembly pathways of OXPHOS complexes, we decided to investigate the impact of different complex III structural defects on the assembly of complex III and the wider OXPHOS system. While both UQCRC1 and UQCR10 are thought to be incorporated into CIII at the same step (Smith et al., 2012;Ndi et al., 2018), as UQCRC1 is required for the dimerization of CIII (Stephan and Ott, 2020) we hypothesized that UQCRC1^KO^ would exhibit a more severe CIII defect, more closely mimicking the loss of MT-CYB (Protasoni et al., 2020) that is known to lead to assembly of the Complex I N-module being blocked. We anticipated that the loss of the small membrane subunit UQCR10 would have minimal impact on CIII assembly, but may lead to the presence of a pool of soluble UQCRC1-UQCRC2 core-module. Both situations where chosen as they were predicted to highlight the utility of SCE-MS to observe aspects of OXPHOS assembly defects not accessible through other techniques. In UQCR10^KO^, while the abundance of complex III subunits were only moderately altered when observed through steady state quantitative proteomics (Figure 3D), SCE-MS showed UQCRC1 and UQCRC2 to increase in solubility (Figure 3F), migrating from a cluster defined by the presence of membrane proteins to one containing predominantly soluble matrix proteins (Figure 3G). Interestingly, we also found that UQCRFS1 is not present in the CIII intermediate found in UQCR10^KO^ that we suspect to be pre-CIII_2_, and like the core module is found predominantly in soluble clusters (Figure 4D). In the high resolution structure of the mature CIII dimer (Gu et al., 2016), the transmembrane domains of UQCRFS1 and UQCR10 sit next to each other. Taken together with our data, this may suggest that UQCR10 plays a direct role in stabilizing UQCRFS1 incorporation into the complex through a coordinated interaction with the translocase BCS1L (Wagener and Neupert, 2012). As expected, UQCRC1^KO^ lacks the later stage CIII intermediates (Figure 3A) and has a greater impact on the steady state abundance of CIII subunits (Figure 4A) than UQCR10^KO^. Loss of UQCRC1 also strongly impacts the abundance of Complex I and blocks its assembly into the supercomplex (Figure 3A). This is unsurprising as assembly of Complex III is known to be critical to the assembly of Complex I (Acin-Perez et al., 2004; Schägger et al., 2004; Protasoni et al., 2020). This was most recently demonstrated using human cybrids lacking MT-CYB, where it was shown through complexome profiling and other techniques that complex I is blocked at the step where the N-module is incorporated (Protasoni et al., 2020). While our steady state proteomic data for the UQCRC1^KO^ showed a decrease in the abundance of N-module subunits (Figures 4A,B) SCE-MS suggested the remaining population trended to being more soluble (Figures 4C,D). We performed complexome profiling on UQCRC1^KO^ to investigate this further, finding multiple holo-Complex I assemblies with and without N-module. Importantly, these results were supported by our in-gel NADH dehydrogenase activity assay (Figure 3C) which confirmed the presence of the N-module on at least one remnant Complex I assembly. Taken together, our data suggests that while Complex I biogenesis strongly relies on the presence of the CIII dimer for efficient assembly, it appears to be able to retain a partial ability to assemble without the structural involvement of CIII. This is not without precedent, as Protasoni and colleagues (2020) also find that 6% of Complex I in their model system remains fully assembled, however unlike in their study we find that the relative amounts of N-module and non-N-module complex I subunits are approximately the same in control and UQCRC1^KO^ cells. Moreover, Protasoni and colleagues show that restoration of the Coenzyme Q pool through expression of an alternative oxidase in their CIII deficient cell line was able to partly restore complex I activity and re-association of the N-module to holo-Complex I. Such an experiment followed by SCE-MS could provide further insights into the nature of N-module association. Possible reasons for the discrepancy between our results and Protasoni et al. (2020) could be cell line specific differences (HEK293 derived from embryonic kidney in our study, and 143B cybrids derived from an osteosarcoma in Protasoni et al., 2020) and resulting differences in the Q-pool, as potential differences in detergent solubilization and BN-PAGE protocols, as well as the nature of the CIII assembly defect. While it is tempting to speculate that the largest confounder is the latter, our UQCRC1^KO^ contains an uncharacterized ∼150 kDa CIII sub-assembly containing MT-CYB, CYC1, UQCRB, UQCRQ and UQCR10 (Figure 3A, Supplementary Figure S4) while their cybrid system harbors no comparable sub-assembly, the ∼150 kDa sub-assembly present in our cell line does not appear to be associated with any Complex I sub-assemblies (Supplementary Figure S4). Thus we conclude that while structural association between Complex III assemblies and the nascent Complex I membrane arm is not required for N-module association, its absence may lead to the interaction being more labile, reflected in an increased susceptibility of N-module subunits to carbonate extraction.

Finally, two additional caveats to SCE-MS introduced by the use of knockout cell lines harboring severe defects in OXPHOS complex assembly should be considered. The first is the potential impact of the carbonate solution on protein complex stability. Since we are directly comparing extractability of proteins from two different complexes, differences in complex stability during the carbonate extraction has the potential to confound results. For example, a sub-assembly unique to a knockout may collapse in the presence of sodium carbonate leading to some subunits being artificially susceptible to extraction from the membrane, whereas the comparable assembly in control cell lines is unaffected. We analyzed membrane fractions from our control and knockout cells extracted at both pH 9.5 and 11.5 using BN-PAGE and immunoblotting (Supplementary Figure S3). This experiment showed that while the susceptibility to extraction changed for some complexes as we expected it would, complexes resistant to carbonate extraction migrated similarly to those solubilized from intact mitochondria. An additional caveat that could be considered is effect of the loss of a protein of interest on mitochondrial lipid composition, which may indirectly alter extractability of proteins. For example, dysfunctional respiratory chain assembly is also known to alter the abundance of cardiolipin (Xu et al., 2019), a mitochondrial specific phospholipid that is critical in the stability of mitochondrial complexes, including OXPHOS complexes (Zhang et al., 2002; Pfeiffer et al., 2003; Mckenzie et al., 2006; Kutik et al., 2008; Gebert et al., 2009). Such alterations could reasonably impact the extractability of complexes between control and knockout cells in a similar way as described above. While measurement of lipid content in our cell lines was out of the scope of our study, we note that while defects in cardiolipin biosynthesis alter the migration and conceivably composition of the pre-sequence translocase of the inner membrane (TIM23 complex) (Kutik et al., 2008), the extractability of Tim23 is not altered (Claypool et al., 2008). Given that the migration of carbonate resistant complexes is not altered in our knockouts (Supplementary Figure S3) we suspect that potential changes in mitochondrial lipid composition would not alter our conclusions.

In summary, SCE-MS is technique that can be used to identify dynamic changes in membrane association on a proteome wide scale. “Destabilization” is an oft-cited concept to describe the impact of an assembly defect on a specific subunit or part of a complex. In most cases it is used speculatively upon observing a reduction in protein abundance, but without other molecular precedent. SCE-MS provides additional insight into “destabilization” by revealing a change in solubility of a protein or population of proteins between two conditions. The quantitative accuracy and high dynamic range of mass-spectrometry relative to techniques such as immunoblotting allow overall reduction in protein abundance to be accounted for, and the global nature of the technique affords the use of 100’s of proteins with known solubility profiles as internal controls. In conclusion, SCE-MS is an easy to implement tool that can add an extra dimension to our understanding of respiratory chain complex assembly.

## Data Availability

The original contributions presented in the study are publicly available. This data can be found here: ProteomeXChange/PXD028935.
