# Supplementary material for: Applying Sodium Carbonate Extraction Mass Spectrometry to Investigate Defects in the Mitochondrial Respiratory Chain
Source: Front Cell Dev Biol. 2022 Mar 1;10:786268. doi: 10.3389/fcell.2022.786268 (PMC8921082; doi:10.3389/fcell.2022.786268)
Supplement: Supplementary file 3 [file DataSheet1.PDF]

Supplementary Material

1.1 Supplementary Figures

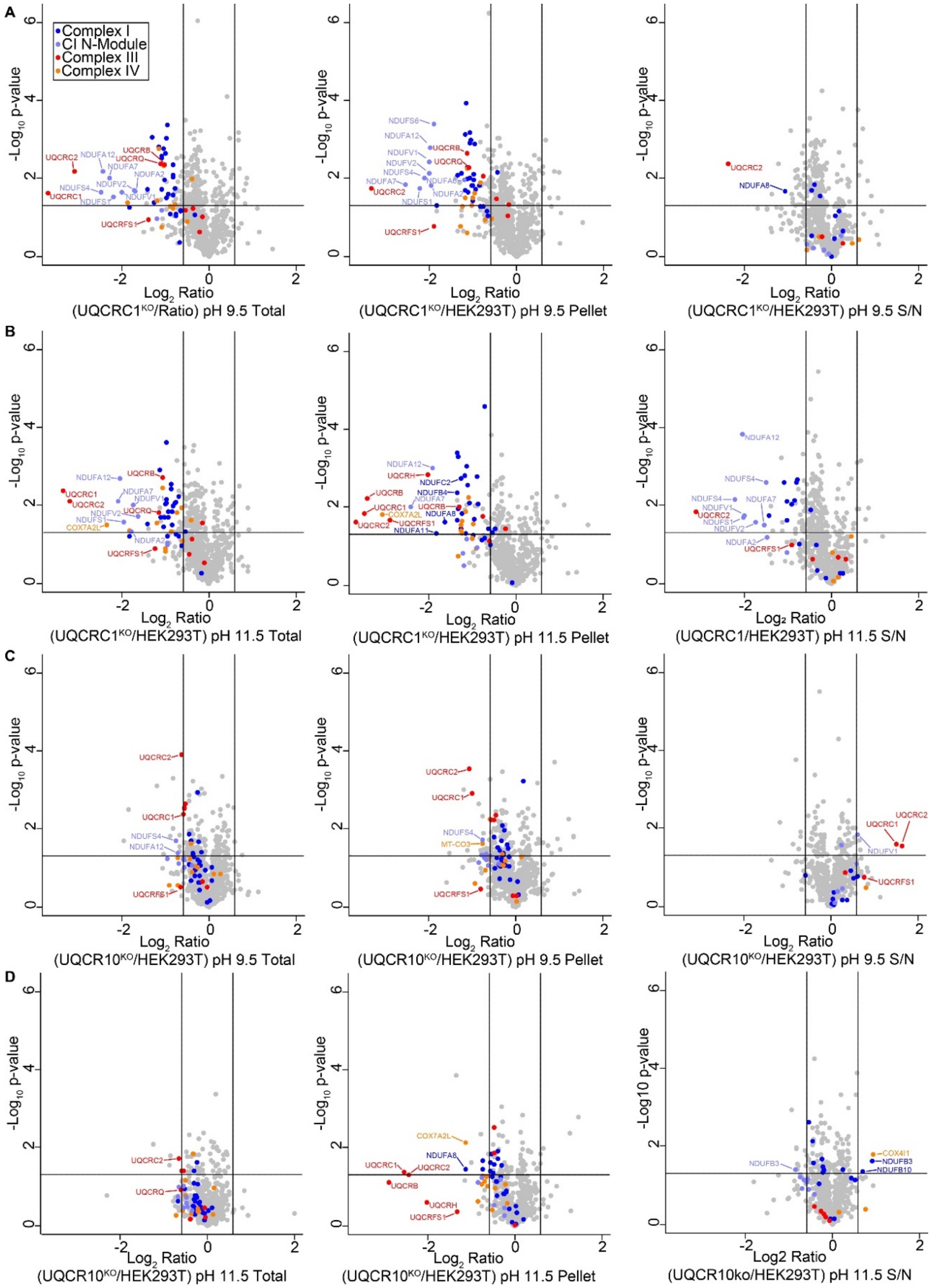

Supplementary Figure 1. Changes in membrane association in CIII knockout cell lines. **A-D**) Volcano plots showing relative abundances of mitochondrial proteins in UQCRC1^{KO} (A-B) or UQCR10^{KO} (C-D) compared to HEK293T control in pellet, supernatant and total fractions at pH 9.5 (A,C) and 11.5 (B,D).

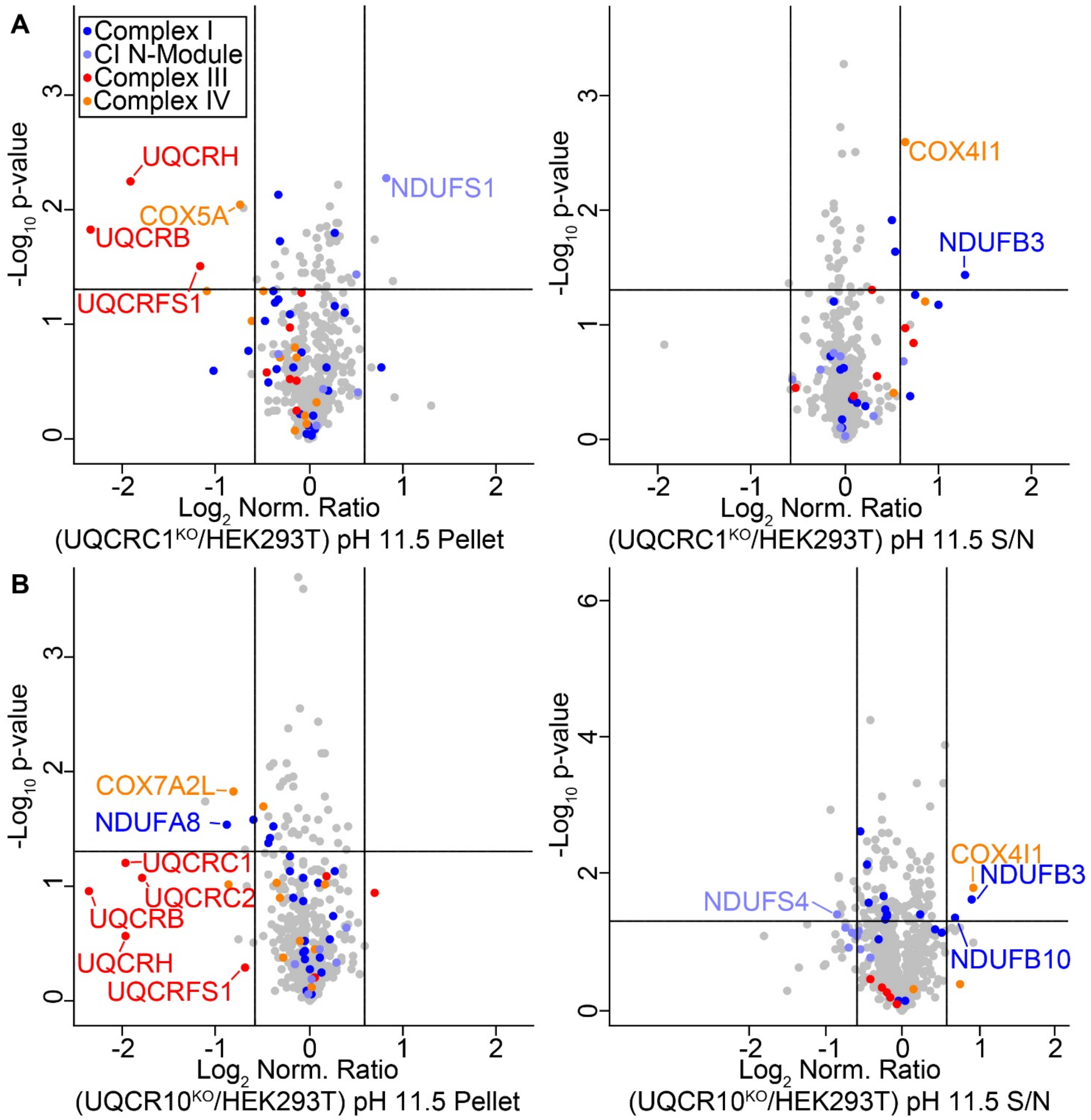

Supplementary Figure 2. Normalized changes in membrane association at pH 11.5. **A-B**) Volcano plots showing relative protein abundance in UQCRC1^{KO} (A) and UQCR10^{KO} (B) pellet and supernatant fractions at pH 11.5 compared to HEK293T, normalized to overall changes of protein abundance in the total fraction.

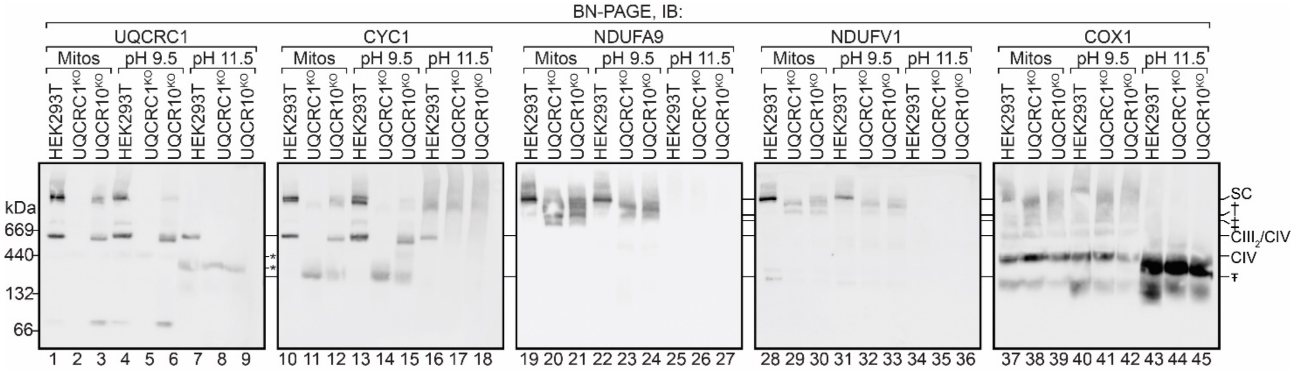

Supplementary Figure 3. Respiratory complexes I, III and IV stay partially intact after sodium carbonate extraction. Mitochondria isolated from the indicated cell lines were either directly solubilized in 1% digitonin (Mitos) or subjected to sodium carbonate extraction at pH 9.5 or 11.5 before the pellet fraction was solubilized in 1% digitonin and analyzed by BN-PAGE and immunoblotting with the indicated antibodies. Non-specific bands marked with *. Ŧ, † and ‡ represent sub-assemblies discussed in text.

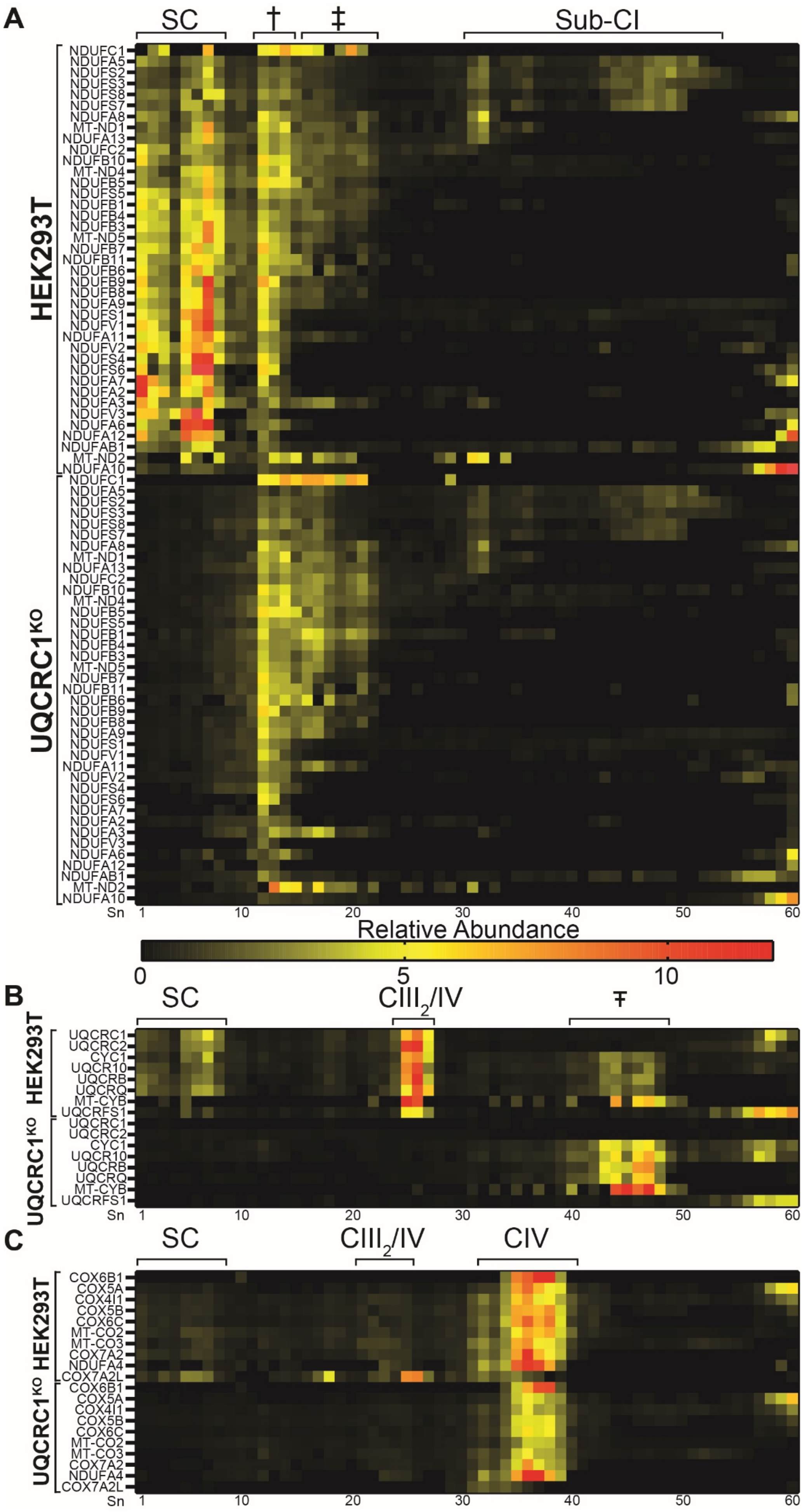
A
SC
†
‡
Sub-CI
HEK293T
UQCRC1KO
NDUFC1
NDUFA5
NDUFS2
NDUFS3
NDUFS8
NDUFS7
NDUFA8
MT-ND1
NDUFA13
NDUFC2
NDUFB10
MT-ND4
NDUFB5
NDUFS5
NDUFB1
NDUFB4
NDUFB3
MT-ND5
NDUFB7
NDUFB11
NDUFB6
NDUFB9
NDUFB8
NDUFA9
NDUFS1
NDUFV1
NDUFA11
NDUFV2
NDUFS4
NDUFS6
NDUFA7
NDUFA2
NDUFA3
NDUFV3
NDUFA6
NDUFA12
NDUFAB1
MT-ND2
NDUFA10
Sn
1
10
20
30
40
50
60
Relative Abundance
0
5
10
B
SC
CIII2/IV
∓
UQCRC1
UQCRC2
CYC1
UQCR10
UQCRB
UQCRQ
MT-CYB
UQCRFS1
C
CIV
COX6B1
COX5A
COX4I1
COX5B
COX6C
MT-CO2
MT-CO3
COX7A2
NDUFA4
COX7A2L

Supplementary Figure 4. UQCRC1^{KO} complexome heatmap. **A-C)** Heatmaps of CI (A), CIII (B) and CIV (C) subunit distribution in UQCRC1^{KO} or HEK293T cells. Values normalized to the HEK293T row average and relative abundances represented as shown in A. Ŧ, † and ‡ represent sub-assemblies discussed in text. Sn, slice number.

1.2 Supplementary Tables

Supplementary Table 1. Sodium carbonate extraction mass spectrometry data (SCE-MS) for control HEK293T mitochondria. SILAC intensities derived from HEK293T samples were processed with the MaxQuant label free quantification (LFQ) algorithm and the results log2 transformed. Pellet and supernatant (S/N) fractions for each pH condition were compared using a two-sided two-sample Student's t-test. The color shading for p-value is set to green for most significant, white for not significant. For fold change, red indicates more abundant, blue less abundant. Mitochondrial (mito) sub-cellular localization annotations are derived from Mitocarta3.0.

Supplementary Table 2. Results from hierarchical clustering of mean LFQ abundance data from Supplementary Table 1. Clusters were defined using the define row cluster tool in Perseus and are indicated in columns K and L. In the color shading for fold change red indicates more abundant, blue less abundant. Mitochondrial (mito) sub-cellular localization annotations are derived from Mitocarta3.0. Secondary tabs detail the contents of each cluster. Cluster 1 contains additional annotations describing the number of predicted transmembrane domains (TMDs) as determined from various algorithms, described in the materials and methods.

Supplementary Table 3. Results from the steady state SILAC quantitative proteomics analysis of UQCRC1 and UQCR10 knockout mitochondria. Mean and individual log2 transformed SILAC ratios and p-values determined through a two-sided single-sample Student's t-test are indicated. The color shading for p-value is set to green for most significant, white for not significant. For fold change, red indicates more abundant, blue less abundant. Mitochondrial (mito) sub-cellular localization annotations are derived from Mitocarta3.0.

Supplementary Table 4. Sodium carbonate extraction mass spectrometry data (SCE-MS) comparing UQCRC1 and UQCR10 knockouts with control HEK293T mitochondria. Log2 transformed SILAC ratios comparing the abundance of proteins in knockout and control total, pellet and supernatant (S/N) fractions for each pH condition were subjected to two-sided one-sample Student's t-tests. The color shading for p-value is set to green for most significant, white for not significant. For fold change, red indicates more abundant, blue less abundant. Mitochondrial (mito) sub-cellular localization annotations and total TMDs are derived from Mitocarta3.0 and Uniprot respectively. Normalized ratios are found in the relevant tab, where the ratio for the total comparison was subtracted from pellet and supernatant ratios as described in the materials and methods and the Student's t-test re-applied.

Supplementary Table 5. Migration profiles of SILAC-labelled UQCRC1knockout and control HEK293T mitochondrial proteins hierarchically clustered using NOVA, listed in MitoCarta 3.0, shown as heatmaps generated from iBAQ values with a colour gradient indicating the min/max for each row (first tab). Entries are curated according to OXPHOS subunits, with all remaining mitochondrial entries identified in the experiment ordered according to hierarchical clustering. For downstream heatmap analysis, individual iBAQ values were normalized by the average wild-type (HEK293T) iBAQ intensity for their respective protein. Complexome mass calibration, using selected protein apparent masses, is shown in the final tab.
